# Aerosol generation during coughing: an observational study

**DOI:** 10.1017/S0022215122001165

**Published:** 2023-04

**Authors:** E Sanmark, L A H Oksanen, N Rantanen, M Lahelma, V-J Anttila, L Lehtonen, A Hyvärinen, A Geneid

**Affiliations:** 1 Facultie of Medicine, University of Helsinki, Helsinki, Finland; 2 Department of Otorhinolaryngology and Phoniatrics – Head and Neck Surgery, Helsinki University Hospital, Helsinki, Finland; 3 Faculties of Science, Mathematics and Statistics, University of Helsinki, Helsinki, Finland; 4 HUS Inflammation Center, Helsinki University Hospital, Helsinki, Finland; 5 HUS Diagnostic Center, HUSLAB, Helsinki University Hospital, Helsinki, Finland; 6 Finnish Meteorological Institute, Helsinki, Finland

**Keywords:** Aerosol Generating Procedure, COVID-19, Cough, Optical Particle Sizer, Aerosol

## Abstract

**Objective:**

Coronavirus disease 2019 has highlighted the lack of knowledge on aerosol exposure during respiratory activity and aerosol-generating procedures. This study sought to determine the aerosol concentrations generated by coughing to better understand, and to set a standard for studying, aerosols generated in medical procedures.

**Methods:**

Aerosol exposure during coughing was measured in 37 healthy volunteers in the operating theatre with an optical particle sizer, from 40 cm, 70 cm and 100 cm distances.

**Results:**

Altogether, 306 volitional and 15 involuntary coughs were measured. No differences between groups were observed.

**Conclusion:**

Many medical procedures are expected to generate aerosols; it is unclear whether they are higher risk than normal respiratory activity. The measured aerosol exposure can be used to determine the risk for significant aerosol generation during medical procedures. Considerable variation of aerosol generation during cough was observed between individuals, but whether cough was volitional or involuntary made no difference to aerosol production.

## Introduction

Airborne transmission is recognised as an important transmission route of severe acute respiratory syndrome coronavirus-2 (SARS-CoV-2), as well as for many other respiratory infections.^1–4^ Aerosol particles are generated during breathing, talking, singing and coughing. They are also presumably generated in higher amounts during certain medical procedures performed in the respiratory tract area, such as otorhinolaryngological and anaesthesiological procedures; these procedures are called aerosol-generating procedures.^5–7^

As the understanding of humans as aerosol generators during normal respiratory activities has increased, the term ‘aerosol-generating behaviours’ has been proposed to be used alongside ‘aerosol-generating procedures’; abandonment of the classification of medical procedures as aerosol-generating procedures has even been proposed.^8^ However, the aerosol-generating procedure classification has been widely used in hospitals globally. Data indicate that surgical procedures involving the mucous membranes and respiratory tract have been postponed during the coronavirus disease 2019 (Covid-19) pandemic for fear of infection.^9^ Thus, variables used for risk assessment in the hospital environment and the area of otorhinolaryngology are still needed.

When assessing the risk of infection, the infectious dose associated with the pathogen, human-related factors such as co-morbidities, the time of exposure and the number of pathogens should be considered.^10^ However, both the infectious dose of different airborne pathogens and the number of infectious pathogens contained in aerosol particles are still widely unknown, and require further investigation before they can reliably be used as part of risk assessment for airborne diseases.^11,12^ As all normal human respiratory activities produce aerosols, simply drawing a line between aerosol-generating and non-aerosol-generating procedures is not sufficient for evaluating the risks of different aerosol exposures in healthcare.^10^ Currently, coughing is assumed to produce a potentially infectious concentration of aerosols, and it has recently been used as a quantitative reference, especially for high-risk aerosol generation during surgery and other medical procedures.^13–16^

In a risk assessment, the amount of aerosol particles that healthcare workers are exposed to is a reasonable observed factor, as the aerosol concentration in an operating theatre dilutes rapidly, especially with highly effective ventilation, which can considerably lower the overall exposure. Therefore, this study aimed to determine the aerosol exposure produced by coughing and thus obtain a scale to compare the aerosol production of other medical procedures in an operating theatre. The comparison of volitional and involuntary coughing allows a broader understanding for cough exposures, as coughing is known to be a heterogeneous activity. The results can be used both to assess the independent risk posed by the cough and, importantly, to produce a reference to evaluate aerosol exposure during potentially aerosol-generating medical procedures such as anaesthesiological and otolaryngological procedures.

## Materials and methods

Particle generation during coughing was measured in 37 volunteers. In addition, involuntary coughs from 15 electively operated patients were measured during local anaesthesia procedures (*n* = 1) and when patients arose from general anaesthesia (*n* = 14). Measurements were conducted in the Helsinki University Hospital's ENT department between December 2020 and February 2021.

The measurements were performed with a TSI® Optical Particle Sizer model 3330, which measures particle size from 0.3 to 10 μm, with a flow rate of 1 litre per minute, and with a measuring interval of 10 seconds. The size range was evaluated to be comprehensive, as 80 per cent to 90 per cent of particles produced during human respiratory activities are smaller than 1 μm after evaporation, and these small aerosols tend to carry most of the pathogens.^17–23^

The operating theatres had a Recair 4C ventilation system with an H14 high-efficiency particulate absorbing filter and ultra-clean ventilation in the laminar area of 1210–1298 litres per second, generating 400–572.83 air changes per hour, meaning a change in total air volume in the operating theatre every 6–10 seconds.

Volitional and involuntary coughing were compared to ensure there was no significant difference between the generated aerosol concentrations, which allowed a more accurate quantitative assessment of volitional coughs. During volitional coughs, the optical particle sizer was positioned at 40 cm, 70 cm and 100 cm from the volunteers, reflecting the same distances and thus the same particle amounts to which medical staff are exposed within the operating theatre. Volunteers were asked to cough as hard as possible towards the optical particle sizer device three to five times from each distance. No additional collection methods, such as funnels, were used to measure the actual particle exposure in a certain spot, considering the rapid spread of aerosols over a wider space.

The recording was continuous, but separate timing of each cough was attempted, thus ensuring that the particles from previous coughs had time to clear from the operating theatre. The marked coughing points were extracted from the continuous measurement data during data analysis, after which they were analysed separately. Not all coughs could be timed as a single cough, considering the sudden, short-term aerosol generation of the cough. In order to ensure that all measurements were as proportional as possible, coughs are shown by cough episodes (i.e. one volunteer and three to five coughs at one distance) in Table 1.

**Table 1.** Observed particle concentration during volitional coughing from different distances

Particle concentration parameter	All volitional coughs	40 cm from source	70 cm from source	100 cm from source
Cough episodes (*n*)	74	37	15	22
Total particle concentration				
– Mean ± SD	1.706 ± 10.802	0.923 ± 5.078	0.091 ± 0.136	4.12 ± 18.771
– Median	0.055	0.055	0.026	0.070
– Range	0.000−88.157	0.013−30.973	0.000−0.466	0.016−88.157
<1 μm particle concentration				
– Mean ± SD	1.692 ± 10.791	0.906 ± 5.030	0.087 ± 0.133	4.111 ± 18.770
– Median	0.044	0.043	0.026	0.053
– Range	0.000−88.141	0.011−30.668	0.000−0.454	0.011−88.141
1−5 μm particle concentration				
– Mean ± SD	0.013 ± 0.035	0.017 ± 0.049	0.004 ± 0.004	0.013 ± 0.006
– Median	0.008	0.008	0.002	0.013
– Range	0.000−0.304	0.000−0.304	0.000−0.012	0.004−0.028
>5 μm particle concentration				
– Mean ± SD	0.001 ± 0.001	0.001 ± 0.001	0.000 ± 0.000	0.001 ± 0.001
– Median	0.000	0.000	0.000	0.001
– Range	0.000−0.005	0.000−0.005	0.000	0.000−0.004

Data represent particles per cubic centimetre, unless indicated otherwise. A cough episode was defined as three to five coughs by one volunteer at a certain distance. Not all volunteers coughed from all distances. Both mean and median values are shown given the large heterogeneity in the data. SD = standard deviation

The involuntary cough measurements were continuous throughout the whole procedure. The times of the coughs were recorded, extracted and analysed. The optical particle sizer was positioned towards the patient, vertically at the patient's head level, at an average of 124 cm (range, 40–180 cm) from the patient, always as close as possible considering the treatment situation. No additional collection methods were used.

As this study combines aerosol physics and medicine, existing power calculators are not available. However, a similar design has been used in a previous study.^15^ As infection risk is related to cumulative aerosol exposure, the mean was calculated for each patient at each coughing distance as a statistical representative. The size-dependent aerosol concentrations measured with the optical particle sizer were normalised with respect to the sizing bin widths within 0.3–10 μm. The particle number size distributions and total particle concentrations per cubic centimetre were calculated. The particles were categorised as follows: smaller than 1 μm, 1–5 μm and larger than 5 μm.

The data were log10 normalised prior to the comparisons. Pairwise comparisons were calculated using the unpaired student's *t*-test with the Benjamini–Yekutieli procedure, with a 5 per cent false discovery rate. The analyses were performed using Excel 2016 spreadsheet software (Microsoft, Redmond, Washington, USA), and GraphPad Prism statistical software version 9.0.2 for Mac (GraphPad Software, San Diego, California, USA) or RStudio version 1.3.959 (R Foundation for Statistical Computing, Vienna, Austria). The minimum concentration in all size classes was 0.000. A *p*-value of less than 0.05 was considered significant.

All procedures that involved human participants were conducted in accordance with the ethical standards of the institutional research committee and the 1964 Declaration of Helsinki. The Ethics Committee of Helsinki University Hospital approved the study protocol (HUS/1701/2020). All participants provided written informed consent prior to their participation.

## Results

Out of 37 volunteers for volitional cough, 28 (76 per cent) were female. The mean age of the volunteers was 41 years (range, 23–61 years). Out of 15 patients examined for involuntary coughing, 8 (53 per cent) were female. The mean age of the patients was 45 years (range, 24–72 years). General anaesthesia was used in 14 procedures with coughing patients and local anaesthesia was used in one procedure.

**Fig. 1.**
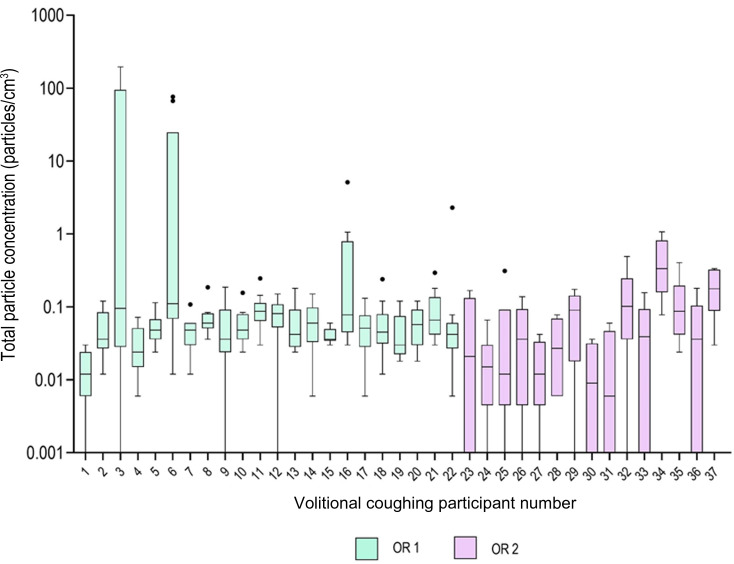
Comparison of volitional coughing between volunteers and different operating rooms (OR), presented as a Tukey box and whiskers plot with outliers. Involuntary coughs could not be combined into the figure, as there was, on average, only one measured involuntary cough per volunteer.

**Fig. 2.**
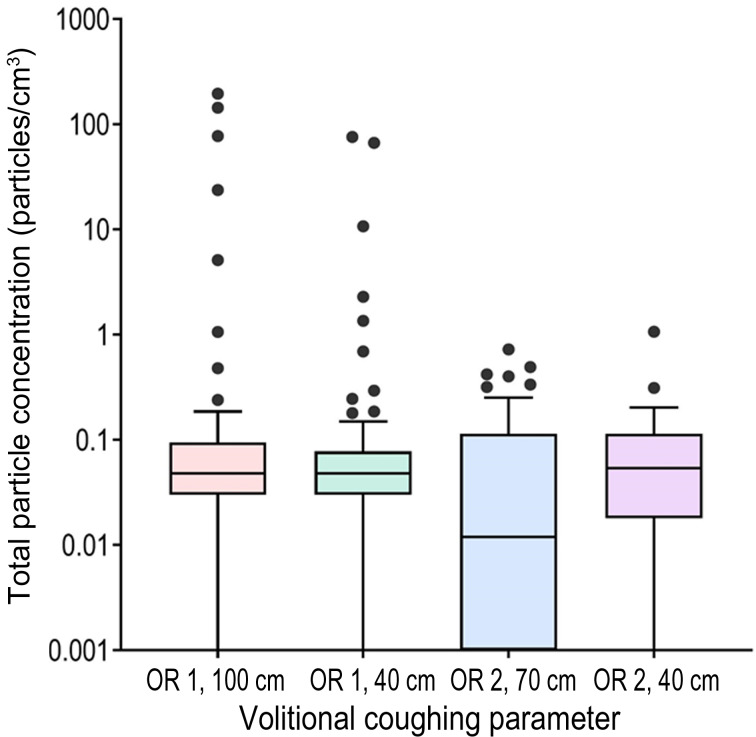
Comparison of volitional coughing between different operating rooms (OR) and at different coughing distances, presented as a Tukey box and whiskers plot with outliers.

A total of 306 coughs were measured from 37 healthy volunteers. Information on particle aerosol concentrations from different distances is presented in Table 1, and compared in Figures 1 and 2. Particle concentrations of 0.000 particles/cm^3^ were measured during 22 of 306 volitional coughs. This reflects not only large differences between individuals in terms of generated concentrations, but also in terms of the dilution and effects of air currents caused by differences in ventilation between the two operating theatres. Background concentrations were low (maximum mean total concentration of 0.0053 particles/cm^3^), which enabled accurate evaluation of particle concentrations generated during coughing.

**Fig. 3.**
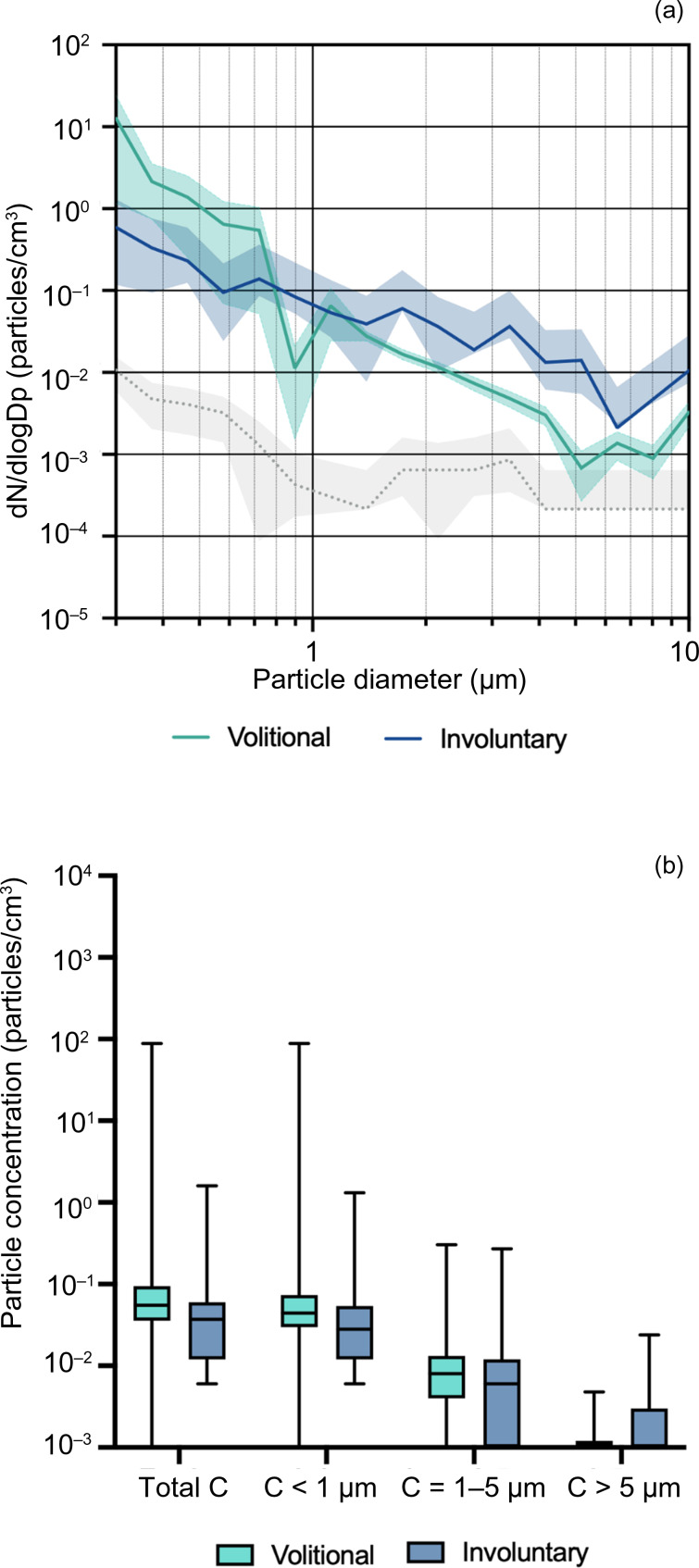
Comparison of volitional coughing versus involuntary coughing. (a) Average aerosol size distributions, presented with background concentration distribution (dotted line), during volitional and involuntary (local anaesthesia) coughs expressed as mean (line) with 95 per cent confidence interval (shaded area). (b) Total concentrations, and concentrations of less than 1 μm, 1–5 μm and more than 5 μm aerosols, during volitional and involuntary coughs, presented as median with interquartile range (box) and range (whiskers). Volitional coughing participants, *n* = 37 (coughs *n* = 306); involuntary coughing participants, *n* = 15 (coughs, *n* = 15). C = concentration; Dp = particle diameter; dN = number of particles; dN/dlogD_p_ = particle size distribution

Mean particle concentration during involuntary coughs was: 0.140 ± 0.332 particles/cm^3^ (range, 0.006–1.308 particles/cm^3^) for particles smaller than 1 μm, 0.025 ± 0.068 particles/cm^3^ (range, 0.000–0.270 particles/cm^3^) for particles 1–5 μm, and 0.002 ± 0.006 particles/cm^3^ (range, 0.000–0.024 particles/cm^3^) for particles larger than 5 μm. There were no significant differences between volitional and involuntary coughing in any particle size category (*p* = 0.244–0.883) (Figure 3).

## Discussion

This study examined aerosol concentration at different distances in volitional and involuntary coughing within the operating theatre, to obtain a perspective on the amount and significance of aerosol generation during medical procedures performed in the operating theatre. We found that the intentionality of coughing did not have a significant effect on aerosol concentration. Rather, large heterogeneity in aerosol generation was observed between individuals. Our results provide systematically collected, distance-scaled, approximate numerical limit values for aerosol exposure encountered by operating theatre personnel that can be used in the risk assessment for aerosol-generating procedures.

During the Covid-19 pandemic, the amount of aerosol produced by medical procedures such as various anaesthetic procedures and otolaryngological surgical procedures has been extensively measured; however, the clinical significance of these procedures from the viewpoint of risk assessment remains unclear compared to aerosol amounts generated in human respiratory activities.^7,15,24^ Our results do not change the fact that there is no absolute quantitative limit for significant aerosol production that poses a risk of infection.^10^ However, in the absence of better understanding, coughing is still commonly used as a limit value for high-risk aerosol output during medical procedures.^15,25,26^ Coughing is an activity that, for example, in the case of ENT diseases, healthcare workers encounter in their work daily, but for a relatively short time.

Comparison of aerosol production in other procedures with coughing helps to categorise the concentration of aerosol generated, such as a lower risk compared to coughing, a similar risk compared to coughing or a higher risk compared to coughing (high-risk aerosol-generating procedure). With this scaling, aerosol production measured in various studies can be brought into a form that can be used and understood clinically: what measures truly exceed aerosol generation of aerosol-generating behaviours? What are the high-risk exposures? On the other hand, these results may aid our understanding regarding exposure to potentially infectious aerosols during epidemics of airborne pathogens and provide useful information to determine the necessary personal protective equipment.^27^ As information on the pathogens contained in aerosol particles – as well as infectious doses of airborne diseases – increases, these factors can later add to the risk assessment of aerosol generation.

The concentrations we measured are consistent with a recent systematic review.^8^ However, our study complements previous studies with exposure-based measurements in the operating theatre and systematic distance-dependent evaluation, which is one of the most significant factors in aerosol exposure.^8,24^ The large range and individual differences of the particle concentrations in our study are seen typically in respiratory activities and related to the heterogeneity of the individual's aerosol generation.^28,29^ However, considering that no difference between volitional and involuntary coughs was observed, and that coughs measured on different days and in different operating theatres are comparable (Figure 2), we conclude that the presented data are representative regarding the exposure of average aerosol concentration generated during coughing. A previous study showed that infected patients generated a greater number of particles when coughing than healthy individuals,^30^ suggesting that the particle concentrations seen in our study represent the minimum value to determine the limit for a high-risk aerosol-generating procedure. In addition, in a clinical context, most patients are not operated on during respiratory tract infection.

In general, distance from the source of infection is a significant measure of exposure. Therefore, we measured cough-produced aerosol concentrations from several different distances.^10^ According to the nature of aerosols, which can travel long distances with air currents, the aerosol concentrations do not necessarily decrease linearly from the source and are influenced by various environmental factors, even in highly ventilated spaces such as operating theatres. This was the case in our study, as the highest aerosol concentrations were observed at a 100 cm distance and the lowest at a 70 cm distance (Table 1, Figure 2). In addition to the nature of the aerosols, this finding can at least partly be attributed to the methodology. The flow rate of the optical particle sizer is 1 litre per minute. When measuring particles with high acceleration at close range, some particles bypass the device and are not recorded. When distance increases further, the acceleration of the particles is reduced, and the concentration is observed more accurately. Still, despite the limitations of the methodology, the optical particle sizer is currently the most suitable and frequently used measuring device in operating theatre conditions.

- Coronavirus disease 2019 highlighted lack of knowledge on aerosol exposure of healthcare personnel during normal patient respiratory activity and suspected aerosol-generating procedures
- Aerosol concentrations generated by volitional and involuntary coughing in the operating theatre were measured
- These concentrations can be used to determine aerosol generation risk during medical procedures
- Whether the cough was intentional or unintentional had no statistically observable effect on aerosol production
- The results provide a reference for assessing and comparing aerosol generation risk during surgical procedures

Other limitations of our study include the different individuals in volitional and involuntary groups, the variable location of the optical particle sizer device during involuntary cough measurements, and the lack of repetitions in the involuntary group.

## Conclusion

This study measured concentrations and size distributions of aerosol particles to which operating theatre personnel are exposed, from volitional and involuntary coughs, at distances typically associated with medical procedures performed in the operating theatre. Whether the cough was intentional or unintentional had no statistically observable effect on aerosol production. These results can be interpreted as a reference for assessing and comparing the risk of aerosol generation during surgical procedures.
